# Real-World Evidence on Low-Dose Olanzapine (≤1.25 mg) for Personalized Antipsychotic Dosing

**DOI:** 10.3390/jpm15080380

**Published:** 2025-08-15

**Authors:** Danbee Kang, Seongmi Moon, Ji-Hyun Baek, Juhee Cho

**Affiliations:** 1 Department of Clinical Research Design and Evaluation, Samsung Advanced Institute for Health Sciences & Technolog (SAIHST), Sungkyunkwan University, Seoul 06351, Republic of Korea; dbee.kang@skku.edu (D.K.);; 2 Center for Clinical Epidemiology, Samsung Medical Center, Sungkyunkwan University School of Medicine, Seoul 06351, Republic of Korea; 3 Department of Psychiatry, Samsung Medical Center, Sungkyunkwan University School of Medicine, Seoul 06351, Republic of Korea

**Keywords:** real-world evidence, low-dose (≤1.25 mg), olanzapine, metabolic safety, personalized psychiatry, individualized dosing

## Abstract

**Background/Objectives**: This cohort study aimed to elucidate the real-world treatment course of patients receiving low-dose olanzapine (<2.5 mg), to assess its efficacy, and to examine its metabolic side effects. This study was a cohort study using a clinical registry. **Methods**: The primary efficacy endpoint was effective medication adherence and appropriate dosing. The primary safety endpoint was the incidence of metabolic adverse events, including diabetes mellitus, dyslipidemia, cardiovascular events, and cerebrovascular events. Cox proportional hazards models were used to compare outcomes between groups. **Results**: A total of 9565 patients were prescribed olanzapine at Samsung Medical Center from 2002 to 2023, and 1629 (17%) were in the low-dose group. The median maintenance period for low-dose olanzapine was 142 days (IQR, 30–551 days), and 95.5% of patients received low-dose olanzapine with either gradual tapering or gradual dose escalation. During follow-up, the risk of diabetes mellitus (HR = 0.32, 95% CI = 0.17–0.62), dyslipidemia (HR = 0.59, 95% CI = 0.42–0.82), cardiovascular disease (HR = 0.88, 95% CI = 0.51–1.49), and cerebrovascular events (HR = 0.75, 95% CI = 0.41–1.36) was lower in the low-dose group than in the regular-dose group. **Conclusions**: Low doses of olanzapine have clinical benefits in providing appropriate dosing and a reduced incidence of metabolic side effects. These findings support personalized antipsychotic treatment strategies, particularly in populations with heightened metabolic vulnerability, by informing dose selection based on individual risk–benefit profiles.

## 1. Introduction

Olanzapine is a thienobenzodiazepine that exerts its effects primarily by blocking dopaminergic and serotonergic receptors [1]. It is indicated for acute and maintenance treatments of schizophrenia, other psychotic disorders, and bipolar disorder [1]. Olanzapine is one of the most frequently prescribed second-generation antipsychotics [2].

It is well established that the efficacy, side effect profiles, and mortality risk of antipsychotic agents are dose-dependent [3]. The use of antipsychotics has been associated with increased cardiac mortality compared to population controls (adjusted relative risk [aRR] 1.83, 95% confidence interval [CI] 1.74–1.93) and antipsychotic-naïve psychiatric patients (aRR 1.72, 95% CI 1.42–2.07), independent of various cardiovascular and psychiatric parameters [4]. Olanzapine is associated with metabolic side effects including fasting glucose and insulin levels, [5,6] diabetes mellitus, dyslipidemia, and other metabolic disturbances [7,8,9]. Consequently, optimizing the clinical benefits of olanzapine, while minimizing its adverse effects, remains a significant challenge for clinicians.

Olanzapine is available in doses ranging from 2.5 mg to 10 mg. Treatment guidelines for adult patients with schizophrenia [10] recommend initiating olanzapine at 5–10 mg with gradual titration up to a maximum of 30 mg, if necessary. For children with schizophrenia, the recommended starting dose is 2.5 mg, with a maximum titration of 20 mg as needed. The typical maintenance dose ranges from 10 to 20 mg, with a defined daily dose (DDD) of 10 mg. Although the most recent treatment guidelines on bipolar disorder [11] no longer provide specific dose recommendations, a similar dosing regimen is often used in clinical practice [12]. However, real-world evidence suggests that lower doses of olanzapine are commonly prescribed, likely because of side effect and patient tolerability concerns. Despite the absence of formal recommendations for doses below 5 mg, a growing interest in low-dose olanzapine (<5 mg) for various conditions exists, particularly in populations that may exhibit heightened sensitivity to standard doses. Notably, East Asian populations have been shown to require different antipsychotic dosing strategies compared to western populations, possibly due to pharmacogenetic and physiological differences [13]. In clinical practice, olanzapine has been prescribed at a wide range of doses including very low doses as low as 1.25 mg/day, particularly in vulnerable populations [14].

Given the increasing real-world use of olanzapine at doses of <2.5 mg, further investigations are warranted to evaluate its prescription patterns, clinical acceptability, and metabolic safety. This study aimed to elucidate the real-world treatment trajectories of patients receiving low-dose olanzapine, assess its effectiveness, and examine its metabolic side effects over an extended follow-up period. This study supports the goals of personalized medicine by providing empirical evidence of dose–response heterogeneity and helping to make tailored prescribing decisions for olanzapine based on patient-specific risk factors.

## 2. Materials and Methods

### 2.1. Study Design and Population

This retrospective cohort study utilized data from the Clinical Data Warehouse (CDW) at the Samsung Medical Center (SMC), covering the period from 1 January 2002 to 31 December 2023. The CDW is a longitudinal database that integrates de-identified patient-level structured and unstructured data extracted from the SMC’s Electronic Health Records (EHRs) through technology-enabled abstraction.

We identified patients who were prescribed olanzapine at least once during the study period (N = 9706). Given that biological differences may influence dose response, we excluded non-Korean patients (N = 141), resulting in a final study sample of 9565 patients. Because the primary objectives of this study were descriptive, power analysis was not required, as all analyses were conducted on pre-existing real-world data. The study received ethical approval from the Institutional Review Board (IRB) of Samsung Medical Center, and the requirement for informed consent was waived because of the use of de-identified data (IRB No. SMC 2024-07-037).

### 2.2. Measurement

From the CDW, we collected demographic, clinical, and medication data for all patients who were prescribed olanzapine. Three experts carefully reviewed and classified diagnoses, medication histories, and treatment patterns.

#### 2.2.1. Definition of Low-Dose and Regular-Dose Groups

Low-dose olanzapine use was defined as a prescription of ≤1.25 mg per day. Patients prescribed at least one low-dose of olanzapine were classified into the low-dose olanzapine group, while all other patients were classified into the regular-dose olanzapine group. The index date was defined as the first date of olanzapine prescription.

Olanzapine prescriptions were classified as on- or off-label based on the ICD-10 diagnostic codes. On-label indications included schizophrenia (F20–F29) and bipolar disorder (F30–F32). Off-label prescriptions were further categorized into dementia, cancer, depression, and other conditions.

#### 2.2.2. Clinical Acceptability and Safety Outcomes

The primary endpoint for clinical acceptability was effective medication adherence and appropriate dosing. Adherence was calculated based on the duration from the first to the last prescription, applying a 30-day grace period to define adherence. A 30-day grace period was applied to account for minor delays in prescription refills before classifying treatment as discontinued. This operational definition is consistent with standard pharmacoepidemiologic practices in observational studies using real-world data [15]. The duration of medication use was calculated from the date of the first prescription to the last recorded prescription, considering any dose adjustments. Adherence to low-dose olanzapine was systematically assessed from the initial prescription to either dose modification or the patient’s last clinical visit. Clinical acceptability was inferred from the use of a gradual tapering strategy or dose maintenance. Gradual tapering involves incremental dose adjustments, either by increasing or decreasing the dose, to manage a patient’s condition without abrupt discontinuation. If a patient maintained the low dose or had only minor dose modifications (increase or decrease) at subsequent visits, this was considered an indication of effective treatment. Conversely, a rapid escalation to the standard therapeutic dose of 10 mg/day at a follow-up visit was classified as a clinical event, suggesting failure in therapeutic management. This escalation was likely prompted by an acute psychotic episode or other severe clinical conditions necessitating a higher therapeutic dose.

The primary safety endpoint was the incidence of metabolic side effects including diabetes mellitus, dyslipidemia, cardiovascular events, and cerebrovascular events. To improve specificity, all events were defined as the co-occurrence of a new ICD-10 diagnosis and a concurrent prescription of disease-specific medication. Diabetes mellitus was defined as new-onset diabetes (ICD-10 codes: E08–E13, E08, E09, E11, and E13) with concurrent anti-diabetic medication use. Dyslipidemia was identified based on the new ICD-10 diagnosis code (E78) and the prescription of dyslipidemia medication. Cardiovascular events were defined using ICD-10 codes I20–I25 and I44–I50 along with the use of cardiovascular medications. Similarly, cerebrovascular events were classified according to ICD-10 codes I60–I69, in combination with cerebrovascular medication use. A detailed list of medications and their classifications is provided in Table S1.

#### 2.2.3. Confounder

Demographic and clinical characteristics were obtained from the date of the first prescription of low-dose olanzapine. Demographic data collected included age, sex, and residential location. Age was categorized into three groups: <18 years, 18–64 years, and ≥65 years. Residential locations were classified as metropolitan or non-metropolitan. Body Mass Index (BMI) was also recorded and categorized as underweight (<17.5), normal (<23.5), overweight (<25), or obese (≥25). Information regarding smoking status (never, former, or current) and alcohol consumption (yes or no) was collected. In addition, baseline laboratory values, including fasting glucose, insulin, HbA1c, HDL cholesterol, LDL cholesterol, triglycerides, AST, and ALT levels, as well as neutrophil percentages, were extracted. Systolic blood pressure was collected as part of routine baseline vital signs at the time of the first olanzapine prescription.

To assess concomitant medication use, all medications co-prescribed with olanzapine were identified and classified by a psychiatry specialist. Psychotropic medications included antidepressants, antipsychotics, and mood stabilizers. Adjuvant treatments for sleep and anxiety disorders included antihistamines, benzodiazepines, and sleeping pills. Medications used to prevent neuropsychiatric side effects included antiparkinsonian drugs and beta-blockers. Other psychiatric-related agents included ADHD medications (stimulants), anxiolytics, and cognitive enhancers (Table S1).

Baseline comorbidities were identified using ICD-10 diagnostic codes recorded up to 1 year before the index date. Comorbid conditions were categorized into neoplasms (ICD-10: C00–C99), endocrine and metabolic diseases (ICD-10: E00–E90), diseases of the nervous system (ICD-10: G00–G99), diseases of the circulatory system (ICD-10: I00–I99), diseases of the respiratory system (ICD-10: J00–J99), and diseases of the digestive system (ICD-10: K00–K93).

### 2.3. Statistical Analysis

Baseline characteristics are reported as mean (standard deviation), number (percentage), or median (interquartile range), as appropriate.

To analyze metabolic adverse events, we excluded patients with pre-existing conditions at the time of their first olanzapine prescription. Patients were followed up from the first date of olanzapine initiation until the occurrence of a study endpoint or the end of their last clinical visit. In this analysis, low- or regular-dose olanzapine was treated as time-varying exposure.

Patients who were initiated on low-dose olanzapine contributed person-times to the low-dose group from the date of initiation until they switched to a regular dose. Similarly, patients who started on a regular dose contributed person-times from the date of initiation until they switched to a low dose. Given the significant confounding effect of age, we used age as a timescale in all models. The incidence of each metabolic outcome was calculated per 10,000 person-years by dividing the total number of events by the cumulative follow-up period. The cumulative incidence of each endpoint was estimated using the Kaplan–Meier method. Hazard ratios (HRs) and 95% CIs were derived using Cox proportional hazards models to compare outcomes between the groups. These models were adjusted for sex, on-label drug use (yes/no), alcohol consumption, smoking status, and BMI (categorized). To minimize data loss, missing values were categorized separately after confirming that they were missing at random. The proportional hazards assumption was verified by visually inspecting the log-minus-log plots and Schoenfeld residuals. All statistical analyses were performed using R version 4.0.3 (R Foundation for Statistical Computing, Vienna, Austria).

## 3. Results

### 3.1. Study Population

A total of 9565 patients were prescribed olanzapine, of whom 1629 (17%) were in the low-dose group. Compared to the regular-dose group, patients in the low-dose group were more likely to be older, female, have a lower BMI, and be non-smokers and non-drinkers.

The prescription of the 2.5 mg olanzapine tablet formulation increased markedly following its market introduction (Figure S1A). However, even before this formulation became available, low-dose olanzapine (≤1.25 mg/day) was already being prescribed by splitting higher-dose tablets. The overall proportion of low-dose use steadily increased throughout the study period (2002–2023) for both on-label (from 0.5% to 19.1%) and off-label (from 3.5% to 28.5%) indications (Figure S1B).

The low-dose group was less likely to use olanzapine for on-label indications (28.8 vs. 42.5% compared to the regular-dose group), particularly for schizophrenia, whereas the use of olanzapine for bipolar disorder was similar between the groups. Among patients taking off-label olanzapine, dementia was the most common indication, followed by cancer. Patients in the low-dose group used significantly fewer antipsychotics (24.4 vs. 37.3%) and benzodiazepines (31.4% and 53.1%) than those in the regular-dose group. Similarly, the use of medications to prevent side effects, such as anti-Parkinson’s drugs, was lower in the low-dose group. In contrast, the low-dose group was more likely to use antidepressants than the regular-dose group (Table 1).

### 3.2. Prescription Patterns

Among the 1629 patients in the low-dose group, 41.1% had previously been on a regular dose before transitioning to a lower dose. After initiating low-dose olanzapine treatment, 50.5% discontinued the medication, whereas 5.0% remained on the low dose for the entire study duration. The remaining 44.5% of the patients increased the dose to a regular dose or that above 10 mg/day. The median maintenance period for low-dose olanzapine was 142 days (IQR, 30–551 days) (Table 2).

The median duration of low-dose olanzapine use was significantly longer for on-label indications than for off-label indications (117 vs. 105 days, *p* < 0.001). Among patients using olanzapine for on-label indications, those who initiated treatment with a low dose had a longer treatment duration than those who started treatment with a regular dose (271 vs. 29 days, *p* < 0.01). Regardless of the indication, 95.5% of the patients received low-dose olanzapine with either gradual tapering or gradual dose escalation, and only 0.5% received rapid dose escalation (Table 2).

### 3.3. Metabolic Side Effects

**Figure 1:**
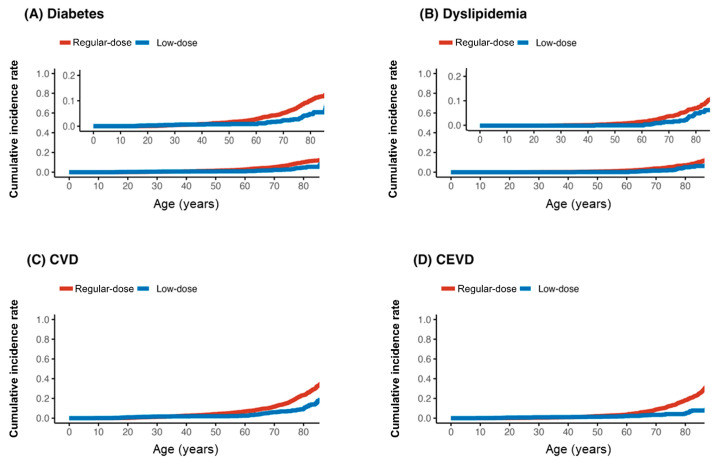
Kaplan–Meier curve for incidence of diabetes (**A**), dyslipidemia (**B**), cardiovascular disease (CVD) (**C**), and cerebrovascular disease (CEVD) (**D**) by low (≤1.25 mg) or regular dose of olanzapine; age was time-axis.

During the follow-up (median follow-up = 2.2, interquartile range = 0.4–5.9 years), the incidence rates per 10,000 person-year of metabolic side effects, including diabetes mellitus (1.7 vs. 4.5), dyslipidemia (8.2 vs. 10.9), cardiovascular diseases (3.7 vs. 3.1), and cerebrovascular events (2.6 vs. 2.3), were generally lower in the low-dose group than in the regular-dose group, respectively (Figure 1).

The risk of diabetes mellitus (HR = 0.32, 95% CI: 0.17–0.62) and dyslipidemia (HR = 0.59, 95% CI: 0.42–0.82) was significantly lower in the low-dose group. The hazard ratios for cardiovascular disease (HR = 0.88, 95% CI: 0.51–1.49) and cerebrovascular events (HR = 0.75, 95% CI: 0.41–1.36) also favored the low-dose group but did not reach statistical significance, likely due to the limited number of events (Table 3).

## 4. Discussion

This study highlighted several key findings regarding the use of low-dose olanzapine. First, the prescription of low-dose olanzapine increased over time for both on- and off-label indications. Second, most patients who were administered low-dose olanzapine followed a gradual tapering or dose escalation strategy, which is considered an appropriate dose adjustment approach, with only 0.5% of patients requiring rapid dose escalation. Third, our results demonstrated a significantly lower incidence of metabolic side effects in the low-dose group than in the regular-dose group.

The increasing off-label use of low-dose olanzapine has been consistently observed in other studies, particularly for the treatment of dementia and cancer. Olanzapine is widely used as a short-term treatment for behavioral disturbances associated with dementia [16]. Several controlled clinical trials have investigated its efficacy and safety in managing the behavioral and psychological symptoms of dementia [16]. More recently, studies have suggested that olanzapine blocks apoE4-catalyzed polymerization of Aβ, showing potential cognitive benefits in Alzheimer’s disease, particularly in APOE_4_ carriers [17]. Apart from psychiatric indications, low-dose olanzapine has gained attention in oncology. The American Society of Clinical Oncology (ASCO) included low-dose olanzapine in its cancer cachexia management guidelines, recommending its use to improve appetite and promote weight gain in patients with advanced cancer [18]. Additionally, low-dose olanzapine has been used in adolescents with anorexia nervosa, highlighting its expanding therapeutic potential beyond that for traditional psychiatric disorders [19,20]. This growing trend underscores an evolving understanding of its therapeutic applications beyond its originally intended uses.

We also found that most patients who received low-dose olanzapine followed a gradual tapering or dose escalation strategy, which is considered an appropriate dose-adjustment approach. Only 0.5% of the patients required rapid dose escalation. A study on dosing strategies for second-generation antipsychotics noted that clinicians often initiate treatment at lower doses and use slower titration rates than package insert recommendations to minimize side effects and improve patient adherence [21]. Additionally, slower tapering of antipsychotics from low doses is associated with lower relapse rates than rapid discontinuation [22]. Clozapine, which shares pharmacological similarities with olanzapine [23], has been recommended at lower and slower titration schedules and reduced maintenance doses, particularly in Asian patients [24]. This aligns with the gradual titration patterns observed in our study, reinforcing the utility of low-dose olanzapine as an effective dose reduction and escalation strategy. Notably, only a few patients required a rapid dose increase owing to clinical events such as relapse, with most patients adequately managed with gradual dose adjustments. The titration and discontinuation patterns observed in this study may reflect the efforts of clinicians to balance efficacy with safety and minimize adverse effects, while maintaining therapeutic goals.

Despite the small sample size, it is notable that 13 patients diagnosed with either bipolar disorder or schizophrenia received low-dose olanzapine as maintenance treatment. These findings suggest that low-dose olanzapine may be suitable for specific patient subgroups, even those with on-label indications, particularly for bipolar disorder. In our cohort, nearly 60% of low-dose users were either adolescents or older adults. These populations are more vulnerable to antipsychotic-related adverse effects, including sedation, weight gain, and metabolic disturbances. For such individuals, reduced dosing may offer a more favorable benefit–risk profile. These findings support the need for dose personalization based on patient-specific factors such as age-related pharmacodynamic sensitivity and tolerability. Although further research is needed to validate these findings and identify which patients are most likely to benefit from long-term low-dose therapy, this approach is consistent with the principles of personalized medicine, which emphasize individualized treatment strategies to optimize both safety and therapeutic effectiveness.

Low-dose olanzapine was also associated with a significantly lower incidence of metabolic side effects than regular-dose olanzapine. These findings align with the existing literature, demonstrating a dose-dependent relationship between olanzapine and metabolic disturbances [25,26]. Lower doses likely reduce the risk of weight gain, insulin resistance, and dyslipidemia, which are commonly attributed to the histaminergic [27] and serotonergic receptor antagonism effects of olanzapine [28]. The 2021 American Psychiatric Association guidelines emphasize that an evidence-based ranking of antipsychotic selection remains challenging owing to significant heterogeneity and limitations in existing data [29]. As a result, medication side effect profiles play a crucial role in treatment decisions, particularly for patients and caregivers concerned with metabolic risks [10]. Our findings highlight the potential of low-dose olanzapine to reduce metabolic burden, supporting its use in patients at higher risk for adverse effects. Despite the increasing real-world use of very low doses (≤1.25 mg) over the past two decades, a dedicated 1.25 mg tablet formulation is currently approved only in Japan (Olanzapine Tablets 1.25 mg “AMEL”). In other countries, including South Korea, clinicians must manually split 2.5 mg tablets to achieve such doses. The growing demand for low-dose strategies underscores the need to consider developing official 1.25 mg tablet formulations to support more flexible and personalized antipsychotic dosing. Overall, these findings highlight the potential of low-dose regimens to mitigate the metabolic burden of olanzapine, making it a viable alternative for patients at an increased risk for metabolic syndrome. Our findings contribute to the growing body of literature that supports applying personalized medicine to psychiatry. The observed variation in treatment duration, response, and adverse outcomes by dose group highlights the importance of individualized prescribing strategies. Real-world dosing data can inform more precise, safe, and effective antipsychotic use, especially in populations with differing pharmacogenetic profiles, such as East Asian patients. Incorporating these insights into clinical decision-making can advance the personalization of psychiatric care.

This study has several limitations. First, the retrospective study design inherently limited the ability to establish causal relationships between low-dose olanzapine and the observed outcomes. Although this study identified important clinical associations, the underlying biological mechanisms driving the dose-dependent effects of olanzapine remain unclear. Second, although adjustments were made for key confounding variables, the possibility of residual confounding factors cannot be excluded. Third, the study did not directly evaluate the effectiveness of low-dose olanzapine on psychiatric symptom control because clinical outcomes such as symptom severity, relapse rates, and functional improvements were not systematically measured. Further research incorporating patient-reported outcomes and clinical assessments is required to confirm the therapeutic efficacy of low-dose olanzapine. Fourth, while the study describes early treatment patterns and short-term outcomes of low-dose olanzapine, we were not able to evaluate the association between treatment duration and long-term clinical outcomes due to the limited number of patients who maintained low-dose therapy over extended periods. Future studies with larger sample sizes are needed to assess exposure–response relationships over time. Lastly, as the study was conducted using data from a single medical center, the findings may not be generalizable to a broader non-Asian population. Future research integrating pharmacokinetic and pharmacogenetic analyses may provide deeper insights into individualized olanzapine dosing strategies and the risk–benefit balance of low-dose regimens.

## 5. Conclusions

In conclusion, this study highlights the increasing use of low-dose olanzapine, both for on- and off-label indications, and its association with a reduced incidence of metabolic side effects. Although a dose of 1.25 mg olanzapine has only been approved in a limited number of countries, our findings highlight its potential clinical utility in managing psychiatric symptoms in patients without formal indications. These results underscore the clinical utility of personalized dosing strategies, promoting safer and more effective antipsychotic use aligned with the principles of individualized medicine. However, given the observational nature of the study, prospective trials are warranted to confirm clinical benefits.

## Figures and Tables

**Table 1:** Baseline characteristics of study population.

	Olanzapine Use		*p*-Value
	Low-Dose (≤1.25 mg) N = 1629	Regular-Dose N = 7936	
Age, years	59.4 (22.9)	51.7 (20.9)	<0.001
<18	105 (6.4)	197 (2.5)	<0.001
18–64	660 (40.5)	5020 (63.3)	
65+	842 (51.7)	2580 (32.5)	
Sex, female	1002 (61.5)	4037 (50.9)	<0.001
Residential area (metropolitan, yes)	1325 (81.3)	6268 (79.0)	0.035
BMI (N = 7326)	23 (6)	23.5 (8)	0.013
Underweight (<17.5)	125 (7.7)	449 (5.7)	<0.001
Normal (<23.5)	676 (41.5)	3072 (38.7)	
Overweight (<25)	209 (12.8)	761 (9.6)	
Obese (≥25)	377 (23.1)	1657 (20.9)	
Unknown	242 (14.9)	1997 (25.2)	
Smoking status			<0.001
Never	1019 (62.6)	2989 (37.7)	
Former	106 (6.5)	315 (4.0)	
Current	123 (7.6)	951 (12.0)	
Unknown	381 (23.4)	3681 (46.4)	
Alcohol consumption			<0.001
No	921 (56.5)	2773 (34.9)	
Yes	282 (17.3)	1345 (16.9)	
Unknown	426 (26.2)	3818 (48.1)	
Systolic blood pressure, mmHg (N = 2418)	123.3 (21.2)	124.7 (21)	0.203
Laboratory finding			
Fasting glucose, mg/dl (N = 7206)	112.6 (43.1)	119.2 (49.2)	<0.001
Insulin level, μIU/mL (N = 776)	15.4 (20.5)	18.5 (39.2)	0.151
HbA1c, % (N = 2276)	6 (1.1)	6.2 (1.4)	<0.001
HDL cholesterol, mg/dL (N = 5106)	56 (16.6)	51.6 (17.4)	<0.001
LDL cholesterol, mg/dL (N = 5083)	100.3 (35.7)	98.8 (37.2)	0.199
Triglyceride, mg/dL (N = 5336)	119.7 (72.5)	119.3 (79.4)	0.872
AST, U/ℓ (N = 7359)	25.8 (31)	31.5 (62.9)	<0.001
ALT, U/ℓ (N = 7359)	22.1 (23.5)	30 (58.7)	<0.001
Neutrophil, % (N = 7342)	59.5 (12.7)	64.4 (15.5)	<0.001
Comorbidities			
Neoplasms	233 (14.3)	1699 (21.4)	<0.001
Endocrine and metabolic diseases	516 (31.7)	1163 (14.7)	<0.001
Diseases of the nervous system	815 (50.0)	1919 (24.2)	<0.001
Diseases of the circulatory system	398 (24.4)	1411 (17.8)	<0.001
Diseases of the respiratory system	126 (7.7)	747 (9.4)	0.036
Diseases of the digestive system	456 (28.0)	1531 (19.3)	<0.001
Olanzapine dose (mg)	1.2 (0.2)	5.3 (3.9)	<0.001
Indication			
On-label	469 (28.8)	3373 (42.5)	<0.001
Schizophrenia	63 (13.4)	1336 (39.6)	<0.001
Bipolar disorder	415 (25.5)	2150 (27.1)	0.190
Off-label	1160 (71.2)	4563 (57.5)	<0.001
Dementia	309 (19.0)	530 (6.7)	<0.001
Cancer	100 (6.1)	1260 (15.9)	<0.001
Depression	91 (5.6)	288 (3.6)	<0.001
Others	683 (41.9)	2630 (33.1)	<0.001
Concomitant medication			
Olanzapine combination			
Antipsychotics	397 (24.4)	2959 (37.3)	<0.001
Antidepressant	853 (52.4)	2148 (27.1)	<0.001
Mood stabilizer	353 (21.7)	1658 (20.9)	0.504
Adjuvant for sleep and anxiety disorders			
Benzodiazepine	512 (31.4)	4211 (53.1)	<0.001
Sleeping pill	57 (3.5)	163 (2.1)	<0.001
Preventing neuropsychiatric side effects			
Antiparkinson drugs	104 (6.4)	791 (10.0)	<0.001
Beta blocking agents	235 (14.4)	998 (12.6)	0.047
Others			
ADHD medication (stimulant)	33 (2.0)	56 (0.7)	<0.001
Anxiolytics	33 (2.0)	81 (1.0)	0.001
Cognitive enhancer	508 (31.2)	633 (8.0)	<0.001
Others	106 (6.5)	938 (11.8)	<0.001

Abbreviations: AST, Aspartate Aminotransferase; ADHD, Attention-Deficit/Hyperactivity Disorder; BMI, Body Mass Index; HbA1c, Glycated Hemoglobin; HDL, High-Density Lipoprotein; LDL, Low-Density Lipoprotein; values were presented n (%), or mean (SD).

**Table 2:** Prescription pattern and clinical acceptability in low-dose olanzapine use.

	Overall (N = 1629)	On-Label (N = 469)	Off-Label (N = 1160)	*p*-Value
First dose (mg)	1.25 (1.25–2.5)	2.5 (1.25–3.75)	1.25 (1.25–2.5)	<0.001
Low-dose (≤1.25 mg)	959 (58.9)	207 (44.1)	752 (64.8)	<0.001
Regular dose	670 (41.1)	262 (55.9)	408 (35.2)	
Duration of low-dose medication (days) *	142 (30–551)	117 (21–662)	105 (30–391)	<0.001
Dose change from low-dose (clinical acceptability) **				<0.001
Stopped	822 (50.5)	190 (40.5)	632 (54.5)	
Maintain	83 (5.1)	13 (2.8)	70 (6.0)	
Increased	716 (44.0)	261 (55.7)	455 (39.2)	
Rapid increased (≥10 mg/day)	8 (0.5)	5 (1.1)	3 (0.3)	
Time to change (months, n = 115)	2.72 (0.44–15.67)	4.26 (0.57–15.57)	1.41 (0.43–16.73)	0.026

* The duration of low-dose medication (days) reflects the total length of time each patient continued low-dose treatment before any dose change, discontinuation, or censoring. ** Dose change from low-dose indicates whether patients maintained, discontinued, or escalated their dose, serving as a proxy for clinical acceptability and tolerability.

**Table 3:** Hazard ratio (95% CI) for incident metabolic side effects.

	Number of Events (Incidence Rate per 10,000 People)		Low vs. Regular (Reference) Crude Hazard Ratio (95% CI)	Low vs. Regular (Reference) Adjusted Hazard Ratio (95% CI)
	Low-Dose (≤1.25 mg)	Regular-Dose		
Diabetes (N = 9199)	10 (1.7)	200 (4.5)	0.28 (0.15–0.53)	0.32 (0.17–0.62)
Dyslipidemia (N = 8226)	39 (8.2)	444 (10.9)	0.54 (0.39–0.75)	0.59 (0.42–0.82)
Cardiovascular diseases (N = 7837)	17 (3.7)	105 (3.1)	0.73 (0.44–1.22)	0.88 (0.51–1.49)
Cerebrovascular diseases (N = 7941)	13 (2.6)	91 (2.3)	0.67 (0.38–1.21)	0.75 (0.41–1.36)

Age as timescale. Adjusted for sex, on-label (yes or no), alcohol consumption, smoking status, and BMI (categorical). Abbreviations: BMI, Body Mass Index; CI, Confidence Interval.

## Data Availability

Data supporting the findings of this study are available from the corresponding author upon reasonable request.

## Supplementary Materials

The following supporting information can be downloaded at: https://www.mdpi.com/article/10.3390/jpm15080380/s1. Figure S1: Annual trends in olanzapine use by dose (A) and proportion of low doses (B). Table S1: Categorized medication.

## Abbreviations

The following abbreviations are used in this manuscript:

ADHD	Attention-Deficit/Hyperactivity Disorder
ALT	Alanine Aminotransferase
AST	Aspartate Aminotransferase
aRR	Adjusted Relative Risk
BMI	Body Mass Index
CDW	Clinical Data Warehouse
CI	Confidence Interval
DDD	Defined Daily Dose
EHRs	Electronic Health Records
HbA1c	Glycated Hemoglobin
HDL	High-Density Lipoprotein
ICD-10	International Classification of Diseases, 10th Revision
IQR	Interquartile Range
IRB	Institutional Review Board
LDL	Low-Density Lipoprotein
RWE	Real-World Evidence
SD	Standard Deviation
SMC	Samsung Medical Center
