# Supplementary material for: Real-World Evidence on Low-Dose Olanzapine (≤1.25 mg) for Personalized Antipsychotic Dosing
Source: J Pers Med. 2025 Aug 15;15(8):380. doi: 10.3390/jpm15080380 (PMC12387202; doi:10.3390/jpm15080380)
Supplement: Supplementary file 1 [file jpm-15-00380-s001.zip › jpm-3742080-supplementary.pdf]

Real-World Evidence on Very Low-Dose (≤1.25 mg) Olanzapine: Prescription Patterns, Clinical Outcomes, and Metabolic Safety

Danbee Kang, Sungmi Moon, Ji-Hyun Baek, Juhee Cho

Table of Contents

(1) Supplementary Tables

(2) Supplementary Figures and Figure Legends

(1) Supplementary Tables

Supplementary Table S1. Medication categorized

Domain	Type of medication	Medication
**Olanzapine Combination**	**Antipsychotics**	Amisulpride, aripiprazole, blonanserin, chlorpromazine, clozapine, haloperidol, paliperidone, perphenazine, pimozide, quetiapine, quetiapine fumarate, quetiapine XR, risperidone, risperidone quicklet, sulpiride, thioridazine, ziprasidone
	**Antidepressant**	Agomelatine, amitriptyline, amitriptyline HCl, amoxapine, bupropion, bupropion HCl, bupropion XL, citalopram, clomipramine, desvenlafaxine, desvenlafaxine SR, duloxetine, duloxetine HCl entericgr., escitalopram, esketamine nasal spray, fluoxetine, fluoxetinedis., fluvoxamine, imipramine, milnacipran, mirtazapine, mirtazapine soltab, moclobemide, nefazodone, nortriptyline, paroxetine, sertraline, tianeptine, tianeptine sodium, trazodone, trazodone hcl, venlafaxine, venlafaxine XR, vortioxetine, wellbutrin
	**Mood Stabilizer**	Carbamazepin CR, carbamazepine, carbamazepine CR, clonazepam, diphenylhydantoin, divalproex, lamotrigine, lithium carbonate, oxcarbazepine, sodium valproate, valproate CR, valproate sodium, valproic sprink
**Adjuvant for sleep and anxiety disorders**	**Benzodiazepine**	Alprazolam, bromazepam, chlordiazepoxide, clobazam, clotiazepam, diazepam, etizolam, flunitrazepam, flurazepam, loflazepate, lorazepam, tofisopam, whanin clonazepam
	**Sleeping pill**	Doxepine, sodium tianeptine, triazolam, eszopiclone, hydroxyzine, melatonin, zaleplon, zolpidem, zolpidem tartrate
**Preventing neuropsychiatric side effects**	**Antiparkinson drugs**	Benztropine, benztropine mesylate, levoda CR(R), madopar dispers., madopar HBS(R), myungdopar(R), perkin(R), pramipexole, pramipexole ER tab., procyclidine, procyclidine HCl, rasagiline, ropinirole, ropinirole PD tab., selegiline HCl, sinemet CR(R), sinemet(R), stalevo(R), trihexyphenidyl, trilevo(R)
	**Beta blocking agents**	Propranolol, propranolol HCl
**Other concomitant medication**	**ADHD medication (stimulant)**	Methylphenidate
	**Anxiolytics**	Buspirone
	**Cognitive Enhancer**	Donepezil, donepezil HCl, donepezil patch, galantamine, galantamine PR, memantine, memantine

		HCl, rivastigmine, rivastigmine patch
	**Others**	Eslicarbazepine acetate, fosphenytoin, gabapentin, lacosamide, levetiracetam, perampanel¸ primidone, rufinamide, tetrabenazine, topiramate, topiramate spr., topiramate XR, vigabatrin, zonisamide
	**Diabetes**	Acarbose, amaryl-M, amaryl-mex(R), apidra, dapagliflozin, dulaglutide, empagliflozin, evogliptin, galvusmet(R), gemigliptin, gemigliptin/metformin, glibenclamide, gliclazide, glimepiride, glipizide, gliquidone, humalog, humalogmix, humulin, humulinn, humulinr, insulatardinnolet, insupenneedle, janumet(R), lantus, levemir, linagliptin, liraglutide, lobeglitazonesulfate, metformin, metformin HCl, metformin XR, mitiglinide, mixtard, nateglinide, novolet, novolin, novomix, novorapid, pioglitazone, repaglinide, rosiglitazone, ryzodeg, saxagliptin, sitagliptin, toujeo, tresiba, vildagliptin, voglibose
	**Dyslipidemia**	Atorvastatin, atorvastatincalcium, atozet(R), caduet(R), ezetimibe, ezetimibe/simvastatin, fenofibrate, fluvastatin, gemfibrozil, litorvazet(R), lovastatin, omega, pitavastatin, pravastatin, rosuvastatin, rosuzet(R), simvastatin, vytorin(R)
**Side effects**	**Cardiovascular diseases**	Carvedilol, carvedilol SR, chlorthalidone, clopidogrel, dabigatran, dalteparin, denopamine, digoxin, diltiazem, diltiazem HCl, diltiazem SR, dipyridamole, dobutamine, dobutamine, dopamine, dronedarone, edoxaban, enalapril, enoxaparin, entresto(R), esmolol, felodipine, flecainide acetate, furosemide, heparin, hydrochlorothiazide, isoket(R)spray, isosorbidedinitrate, isosorbide mono, ivabradine, l-carnitine, metolazone, metoprolol, milrinone, molsidomine, nadolol, nadroparine, nebivolol, nicorandil, nifedipine, nifedipine, nitroglycerin, perindopril, perindopril arginine, prasugrel, propafenone, propafenone HCl, ramipril, rivaroxaban, sotalolhcl, spironolactone, ticagrelor, ticlopidine, tolvaptan, torasemide, tparecombinant, trimetazidine, trimetazidine HCl, ubidecarenone, urokinase, verapamil, warfarin, yuclid tab(R)
	**Cerebrovascular diseases**	Argatroban, clopidogrel, heparin, nimodipine, ticagrelor, ticlopidine, TPA recombinant, urokinase, yuclid tab (R)

(2) Supplementary Figures and Figure Legends

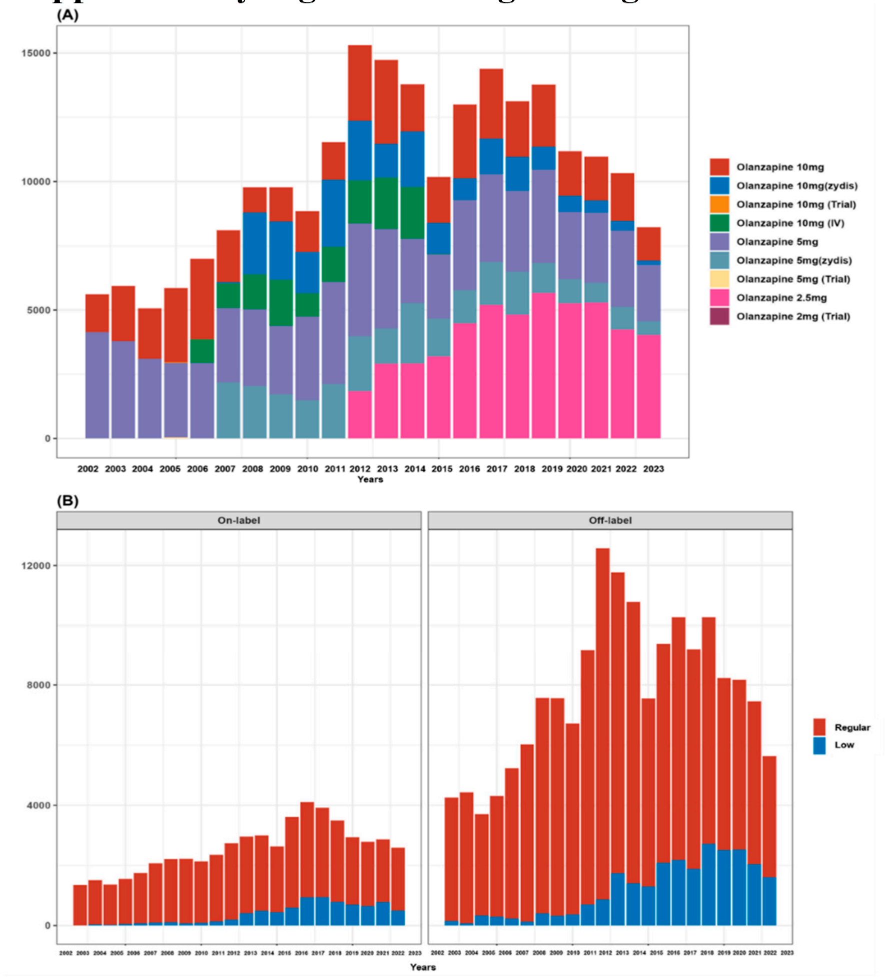

Supplementary Figure S1. Annual trends in olanzapine use by dose (A) and proportion of low doses (≤1.25 mg) (B)
